# Nuclear Abnormalities in *LMNA* p.(Glu2Lys) Variant Segregating with *LMNA*-Associated Cardiocutaneous Progeria Syndrome

**DOI:** 10.3390/genes15010112

**Published:** 2024-01-18

**Authors:** Matheus V. M. B. Wilke, Myra Wick, Tanya L. Schwab, Rodrigo Tzovenos Starosta, Karl J. Clark, Heidi M. Connolly, Eric W. Klee

**Affiliations:** 1 Center for Individualized Medicine, Mayo Clinic, Rochester, MN 55905, USA; wilke.matheus@mayo.edu; 2 Department of Obstetrics and Gynecology, Mayo Clinic, Rochester, MN 55905, USA; wick.myra@mayo.edu; 3 Department of Clinical Genomics, Mayo Clinic, Rochester, MN 55905, USA; 4 Department of Molecular Hematology, Mayo Clinic, Rochester, MN 55905, USA; 5 Division of Medical Genetics and Genomics, Washington University in Saint Louis, Saint Louis, MO 63130, USA; rodrigo.starosta@cuanschutz.edu; 6 Graduate Program in Gastroenterology and Hepatology, Universidade Federal do Rio Grande do Sul, Porto Alegre 90610-000, Brazil; 7 Department of Biochemical and Molecular Biology, Mayo Clinic, Rochester, MN 55905, USA; 8 Department of Cardiology, Mayo Clinic, Rochester, MN 55905, USA; 9 Department of Quantitative Health Sciences, Mayo Clinic, Rochester, MN 55905, USA

**Keywords:** atypical progeroid syndrome, *LMNA*, mitral valve calcification, nuclear abnormalities, case report

## Abstract

The *LMNA* gene encodes lamin A and lamin C, which play important roles in nuclear organization. Pathogenic variants in *LMNA* cause laminopathies, a group of disorders with diverse phenotypes. There are two main groups of disease-causing variants: missense variants affecting dimerization and intermolecular interactions, and heterozygous substitutions activating cryptic splice sites. These variants lead to different disorders, such as dilated cardiomyopathy and Hutchinson–Gilford progeria (HGP). Among these, the phenotypic terms for *LMNA*-associated cardiocutaneous progeria syndrome (LCPS), which does not alter lamin A processing and has an older age of onset, have been described. Here, we present the workup of an *LMNA* variant of uncertain significance, NM_170707.2 c. 4G>A, p.(Glu2Lys), in a 36-year-old female with severe calcific aortic stenosis, a calcified mitral valve, premature aging, and a family history of similar symptoms. Due to the uncertainty of in silico predictions for this variant, an assessment of nuclear morphology was performed using the immunocytochemistry of stable cell lines to indicate whether the p.(Glu2Lys) had a similar pathogenic mechanism as a previously described pathogenic variant associated with LCPS, p.Asp300Gly. Indirect immunofluorescence analysis of nuclei from stable cell lines showed abnormal morphology, including lobulation and occasional ringed nuclei. Relative to the controls, p.Glu2Lys and p.Asp300Gly nuclei had significantly (*p* < 0.001) smaller average nuclear areas than controls (mean = 0.10 units, SD = 0.06 for p.Glu2Lys; and mean = 0.09 units, SD = 0.05 for p.Asp300Gly versus mean = 0.12, SD = 0.05 for WT). After functional studies and segregation studies, this variant was upgraded to likely pathogenic. In summary, our findings suggest that p.Glu2Lys impacts nuclear morphology in a manner comparable to what was observed in p.Asp300Gly cells, indicating that the variant is the likely cause of the LCPS segregating within this family.

## 1. Background

The *LMNA* gene encodes both lamin A and lamin C, which have crucial structural and regulatory roles in nuclear organization, being present in the nuclear lamina beneath the inner nuclear membrane [1,2]. Pathogenic variants in the *LMNA* gene are responsible for laminopathies, a group of disorders with diverse phenotypes ranging from the severe and systemic Hutchinson–Gilford progeria (HGP, OMIM 176670) to more tissue-specific dilated cardiomyopathy (OMIM 115200) [3].

Two major groups of disease-causing variants have been associated with *LMNA*. The first group comprises missense variants that affect the dimerization of lamin A/C and/or intermolecular interactions in the nuclear lamina, leading to isolated disorders such as dilated cardiomyopathy or some progeroid syndromes. The second group comprises heterozygous substitutions at the junction of exon 11 and intron 11, which activate cryptic splice sites, leading to in-frame deletions, including the proteolytic site required for the maturation of lamin A. This results in the production of progerin, a mutant form of lamin A responsible for HGP [4]. The mechanisms and effects of most *LMNA* variants causing laminopathies are not well understood, including their association with specific phenotypes [3]. Two hypotheses have been proposed to explain laminopathy development: one suggests structural and mechanical changes, such as abnormal nuclear rigidity and shape, while the other focuses on functional abnormalities related to changes in gene expression patterns. The second hypothesis relates to the association between the nuclear lamina and lamina-associated domains (LADs), which are involved in genome regulation [3,5,6].

More than 790 *LMNA* variants have been described in the Human Gene Mutation Database (HGMD; date of accession: February 2023) associated with over 200 different reported phenotypes that overlap with one another. Among these is the phenotype “atypical progeria syndrome” (APS), which is associated with an older age of onset and genetic alterations that do not alter lamin A processing [4,7,8].

Here, we report the case of an individual with atypical progeria and features of *LMNA*-associated cardiocutaneous progeria syndrome (LCPS) who has a positive family history of similar symptoms segregating with the variant LMNA:c.4G>A (p.Glu2Lys). As the proband’s family was previously published with the eponym of Lesser syndrome, we further characterized this LCPS phenotype via functional testing to assess nuclear morphology by transfection and overexpression of *LMNA*-expressing constructs [9]. Further characterization of the atypical forms of *LMNA*-linked progeria is important to better understand the clinical course of these patients.

## 2. Methods

### 2.1. LMNA Plasmid Construction

The vector Lamin A-mRFP, containing the *LMNA* wild-type (WT) cDNA, was received as a gift from Eric Schirmer (Addgene, Plasmid No. 124268). First, Lamin A-mRFP was linearized using XhoI/AscI, removing an 81 bp fragment (backbone 6581 bp). Oligo 1 (Table 1) was then digested using XhoI/AscI, also producing an 81 bp fragment, and ligated to the backbone (6581 bp) of Lamin A-mRFP, producing the Lamin A-mRFP-K (WT) construct. A second construct was made by using the same method with Oligo 2 (Table 1) to introduce the *LMNA* p.(Glu2Lys) variant, producing the Lamin A-mRFP-E2K-K construct. In order to produce the *LMNA* p.(Asp300Gly) variant, Lamin A-mRFP-K was linearized using BlpI, removing a 600 bp product (backbone 6062 bp). Oligo 3 (Table 1) was also digested using BlpI, producing a 600 bp product that was ligated to the backbone (6062 bp) of Lamin A-mRFP-K, producing the Lamin A-mRFP-D300G-K construct. The Lamin A-mRFP-K (WT), E2K, and D300G constructs were then subcloned into the pcDNA5-FRT-TO (Invitrogen, Waltham, MA, USA) vector for the generation of stable cell lines. First, Lamin A-mRFP-K (WT) was digested using BamH1/NotI, producing a 2670 bp fragment containing Lamin A-mRFP. pcDNA5-FRT-TO was then digested with BamH1/NotI, removing a 50 bp fragment (backbone 5087 bp), and the 2670 bp Lamin A-mRFP fragment and the pcDNA5-FRT-TO backbone (5087 bp) were ligated, producing the pcDNA5-FRT-TO-Lamin A-mRFP-K (WT) construct. The same method was used to generate both the pcDNA5-FRT-TO-Lamin A-mRFP-E2K-K and pcDNA5-FRT-TO-Lamin A-mRFP-D300G-K constructs. All plasmids were Sanger sequence confirmed to ensure incorporation of the appropriate gene variation.

### 2.2. LMNA Stable Cell Lines

*LMNA*-mRFP stable cell lines were generated from all three constructs using the Flp-In T-Rex Core Kit (Invitrogen). Briefly, Flp-In T-Rex 293 cells (Invitrogen) were transfected with the pOG44 plasmid and the appropriate pDNA5-FRT-TO-Lamin A-mRFP-K construct using Lipofectamine 3000 (Thermo Fisher Scientific, Waltham, MA, USA). Forty-eight hours post-transfection, cells were passed into media containing 150 ug/mL hygromycin (Roche, Basel, Switzerland) for selection. Approximately two weeks later, single colonies were isolated and then expanded to make stable cell lines. The lines were then screened to confirm expression of the *LMNA* protein by mRFP expression after doxyclycline induction using immunocytochemistry (described in the next section).

### 2.3. Immunocytochemistry and Nuclear Morphology Assessment

Assessment of nuclear morphology was performed using the immunocytochemistry of stable cell lines expressing *LMNA*-mRFP WT, E2K, and D300G at four days post-induction of protein expression. Briefly, *LMNA*-mRFP stable cell lines (WT, p.E2K, p.D300G) were plated in 6-well plates containing glass slides at 2 × 10^5^ cells/well and incubated at 37 °C O/N. Cells were then induced with 1 μg/mL doxycycline for a total of 4 days. At 4 days post-induction, slides were fixed with 4% paraformaldehyde, mounted with Vectashield containing DAPI (Vector Laboratories Inc., Newark, CA, USA), and mRFP fluorescence was imaged using an LSM980 Airyscan 2 confocal microscope (Zeiss, Jena, Germany) to assess the impact on nuclear morphology.

### 2.4. ImageJ

The levels of Lamin A/C at the nucleus were analyzed using ImageJ software (National Institutes of Health, Bethesda, MD, USA), as described previously [10]. All morphometric processing and analysis were performed by the author (RTS), who was blinded as to group (patient or control). Images were obtained in Tag Image File Format (.tiff) in RGB color mode and initially converted to 8-bit. Juxtaposed nuclei were manually separated, and overlapping nuclei were delineated when possible with the exclusion of the underlying nucleus; when delineation of overlapping nuclei was not possible, such nuclei were excluded. Automatic thresholding of the 8-bit images was performed, with small (<10%) manual adjustments to maximize definition. Particles were analyzed with the following constraints: minimum size of 10 pixels, circularity of 0.1–1.0, and exclusion of particles touching the image edge.

### 2.5. Statistical Analysis

Statistical analysis was performed using analysis of variance (ANOVA) between the groups p.(Glu2Lys), p.Asp300Gly, and WT for the ImageJ variables AREA, PERIM, and FERET. Effect sizes were calculated using η^2^ and η^2^p. Additionally, post hoc tests with Tukey correction were performed to assess which groups differed. *p*-values below 0.05 were considered significant. All analyses were conducted using JASP version 0.16.3.0 [11].

## 3. Results

### 3.1. Case Presentation

The proband is a 38-year-old white female with a medical history characterized by severe calcific aortic stenosis, calcified mitral valve, premature aging, and a family history of similar features. Her early development was, reportedly, unremarkable. The patient reported experiencing greying of her hair in her 20s, followed by symptoms of progressive fatigue and exertional dyspnea. She received regular cardiovascular follow-up locally, where she was seen by the same cardiologist as several family members. At the age of 35, the patient was referred to Mayo Clinic for management of her symptomatic valve disease.

The patient’s clinical phenotype included a low BMI (16.7), with her height being 173 cm and weight being 50 kg. Her echocardiogram demonstrated severe aortic stenosis with a mean gradient of 51 mmHg, moderate mitral stenosis with a mean gradient of 6 mmHg, and mild-to-moderate mitral regurgitation. Transcatheter aortic valve replacement was considered, but preoperative computerized tomography (CT) showed extensive calcification of the aortic valve, extending into the left ventricular outflow tract and the anterior leaflet of the mitral valve. No significant coronary artery disease was observed by CT. The patient underwent both mitral and aortic valve replacement, tricuspid valve repair, and patent foramen ovale closure at the age of 35. There was no evidence of lipodystrophy, muscle weakness, or cutaneous findings.

**Figure 1:**
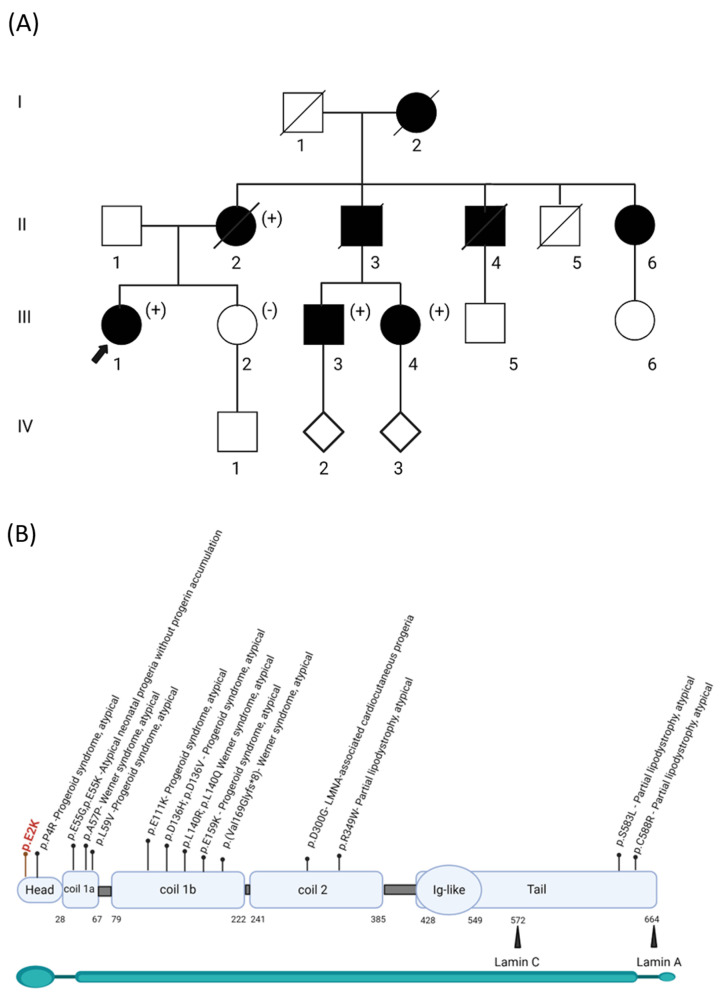
Segregation of the p.(Glu2Lys) variant in the proband’s family and its location in the *LMNA* protein. (**A**) Pedigree of the family, showing the segregation of the *LMNA* variant as either positive (+) or negative (-). Affected individuals with aortic and mitral valve calcifications are represented by black squares/circles. The proband is indicated by an arrow. I–V refers to the e generation number in the family. (**B**) Distribution of the variants described in HGMD across different domains that are associated with the atypical phenotype. The start and end residues of the domains are represented as amino acid numbers.

The family history was positive for multiple family members reported to have similar phenotypes, including her grandmother, mother, and uncles (Figure 1A). Her maternal grandmother (individual I.2) passed away at the age of 32, and an autopsy showed a heavily calcified mitral valve and aortic valve with friable verrucous vegetations on both valves. Genetic testing by Sanger DNA sequencing for a subset of genes associated with HGP was performed on some affected family members previously, which reported a variant of uncertain significance in *LMNA* NM_170707.2 c. 4G>A, p.(Glu2Lys). However, the proband was not included in this testing. She subsequently underwent clinical exome sequence analysis at the age of 35 by GeneDx, which reported the same *LMNA* variant. This variant was initially classified as a Variant of Uncertain Significance (VUS) because it was absent from the gnomAD database (PM2_supp), and in silico predictions indicated uncertainty (REVEL 0.35). To better assess the role of this variant and fulfill the ACMG criteria for functional studies, PS3 in vitro studies were conducted, as reported below [12]. The variant was further classified as likely pathogenic, supported by segregation studies (PP1_strong, PM2_supp, PP4, and PS3_supp).

### 3.2. Nuclear Morphology

**Figure 2:**
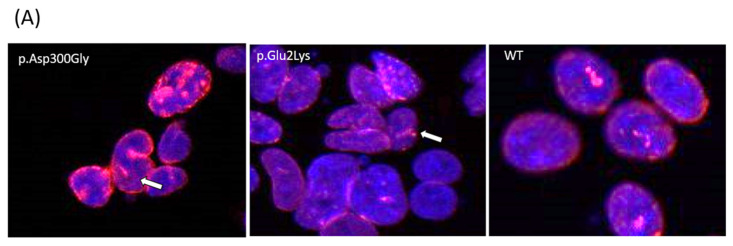

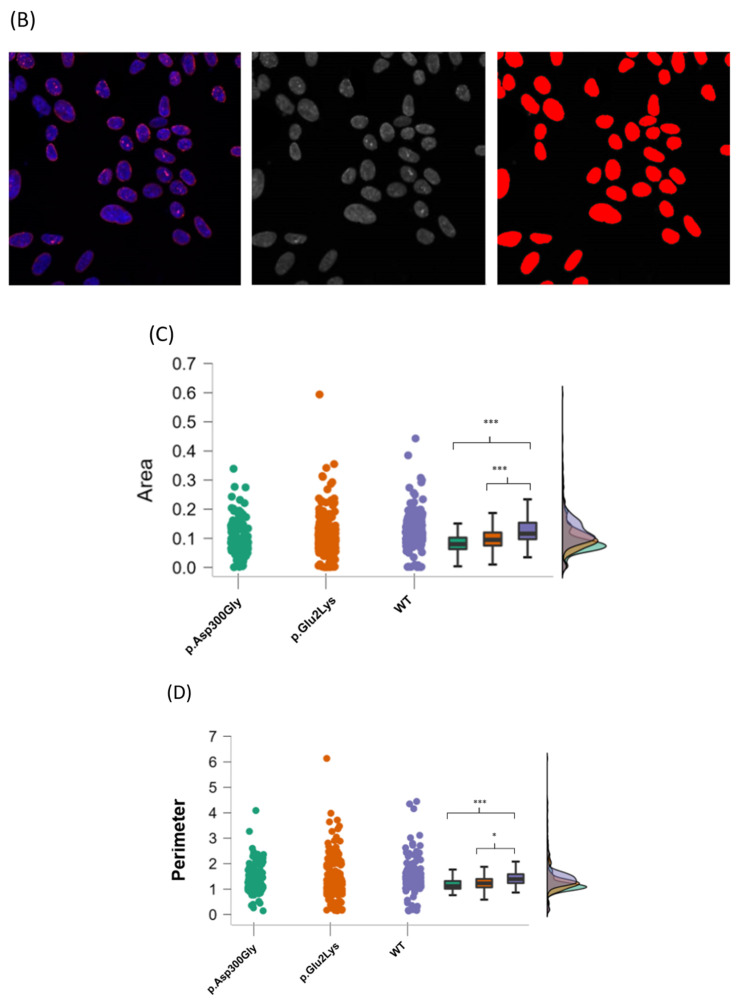

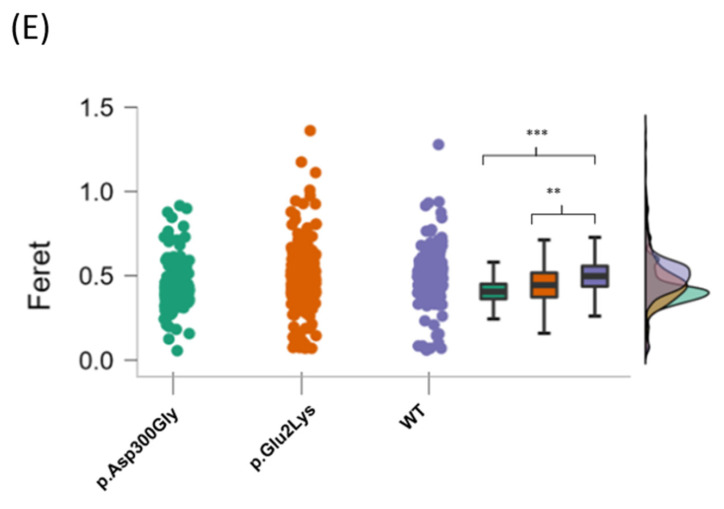
Nuclear abnormalities in p.Glu2Lys cells. (**A**) Indirect immunofluorescence analysis of nuclei shape from a positive control (p.Asp300Gly), the proband’s variant (p.Glu2Lys), and a negative control (WT). White arrows indicate nuclei lobulations. (**B**) ImageJ analyses of nuclear morphology. (**C**–**E**) Raincloud Plot of the analyzed variables between a positive control p.Asp300Gly, our proband’s variant p.Glu2Lys, and WT. * *p* < 0.05, ** *p* < 0.01, *** *p*< 0.001.

Indirect immunofluorescence analysis of nuclei from stable cell lines showed abnormal morphology, including lobulation and occasional ringed nuclei, as previously described for the p.Asp300Gly variant (Figure 2A).

Additionally, nuclear images were analyzed with the ImageJ software (version 1.53v 21 November 2022) using three parameters: area, perimeter, and Feret’s diameter (also known as maximum caliper). In total, 175 nuclear objects from the p.Asp300Gly variant, 326 from the p.Glu2Lys variant, and 270 from WT nuclei were evaluated (Figure 2B).

Relative to the controls, p.Glu2Lys and p.Asp300Gly nuclei had significantly (*p* < 0.001) smaller average nuclear areas than controls (mean = 0.10 units, SD = 0.06 for p.Glu2Lys and mean = 0.09 units, SD = 0.05 for p.Asp300Gly versus mean = 0.12, SD = 0.05 for WT).

Regarding nuclei perimeter, a significant difference was found between the p.Glu2Lys and WT groups (mean = 1.34 units SD = 0.6 versus mean = 1.44 SD = 0.5, *p* < 0.05). Nuclei perimeter was also smaller in p.Asp300Gly compared to WT (mean = 1.2 units, SD = 0.49 versus mean = 1.44 units, SD = 0.5, *p* < 0.001).

Significant differences were found in the Feret diameter between p.Asp300Gly and WT (mean = 0.43 units, SD = 0.13, versus mean = 0.49 units, SD = 0.13, *p* < 0.001) and between p.Glu2Lys (mean = 0.46 units, SD = 0.16) and WT (*p* < 0.01). No significant difference was found between the nuclei from p.Glu2Lys and p.Asp300Gly in any of the evaluated parameters. Figure 2C–E shows the raincloud plots for the analyzed variables.

## 4. Discussion

The *LMNA* protein consists of several domains, including a central α-helical rod domain, a carboxy-terminal globular domain, and a variable amino-terminal domain [2,3,4]. Lamin A, but not lamin C, has a C-terminal tail end that undergoes many post-translational modifications for the maturation from prelamin A to lamin A [4]. APS is not clearly defined in the literature, but it is characterized by different clinical presentations than expected for classical laminopathies and does not alter the lamin A processing [8]. Previous studies have shown that in skin fibroblasts isolated from individuals with APS, the processing and levels of lamin A and C are normal, unlike those with a variant associated with HGPS [7]. No accumulation of abnormal species of lamins A or C and the appearance of several deformed nuclei (including multilobulations and nuclear membrane invagination) were reported in the skin cells from a cohort of patients with an atypical progeria syndrome reported by Garg et al. in 2009 [8]. According to HGMD, 14 different variants of the *LMNA* gene have been reported to be associated with “atypical” laminopathies, spreading over all the domains with some hotspot regions such as the amino acids Gln55, Asp136, and Leu140, as demonstrated in Figure 1B.

The p.Glu2Lys variant is absent from both the gnomAD and HGMD databases and is located in a conserved amino acid in the N-terminal head region of the lamin A/C protein. The *LMNA* head seems to facilitate the formation of longitudinal polar head-to-tail polymers through the promotion of secondary structural organization in lamin parallel coiled-coil homodimers [8,13]. The p.Glu2Lys variant results in the substitution of an acidic amino acid, glutamine, with a basic amino acid, lysine, having a REVEL score of 0.35 (uncertain). Due to the in silico uncertainty of the variant, our study aimed to pursue supporting evidence of pathogenicity for this variant. The method to assess LMNA pathogenicity has become a topic of recent debate in the literature. Variants in LMNA are implicated in more than a dozen diverse Mendelian disorders, and evaluating their functional effects poses challenges due to the gene’s pleiotropy and potential involvement in various pathogenic mechanisms [14]. Notably, many LMNA variants do not lead to the typical accumulation of progerin, as seen in Hutchinson–Gilford progeria syndrome [15]. Recent work, focusing on myopathic lamin missense variants, assessed 178 missense variants employing human embryonic kidney (HEK) 293 cells and mouse C2C12 myoblasts. The author used nuclear aggregation as a key functional output and advocated that this would be a scalable study to support the pathogenicity of these variants [16].

In our study, we observed that cells from our proband with the p.(Glu2Lys) variant were significantly smaller in both area and perimeter compared to the wild type, similar to cells transfected with the p.Asp300Gly variant demonstrating good cellular phenotypic overlap being used as supporting evidence of pathogenicity to assess the variant (PS3_supp). The phenotype associated with the p.Asp300Gly variant is also very similar to our case: the proband was normal throughout childhood and young adult life and is described to have developed taut skin, loss of subcutaneous fat stores, thinning and graying of scalp hair, and a prematurely aged appearance around the age of 20 [7]. Exertional dyspnea started at the age of 27, and subsequently, mitral annular calcification, mitral regurgitation, and aortic valve stenosis were noted [7]. Patients with ATS segregating with the p.Pro4Arg variant were nicely summarized in another paper that demonstrated conflicting clinical overlap: some of the cases presented metabolic abnormalities such as insulin resistance and lipodystrophy at a younger age while others did not [17].

This variant was previously described in a case report in the MAG Journal as a variant of uncertain significance in a patient with Lesser Syndrome, who presented with premature aging and valve calcifications [9]. In the present case, we found the variant segregating in four symptomatic individuals (proband’s mother, two cousins, and the proband) and absent in one asymptomatic individual, which allowed us to upgrade its classification using PP1_Strong due to the number of meiosis as previously described [18]. We suggest that the eponym Lesser syndrome should be replaced by the term LCPS-like syndrome because of the similarities shared with the proband of Kane et al. paper 2013 [7].

One of the hallmarks of LCPS seems to be the marked calcification of cardiac structures, mainly the mitral and aortic valves; however, it is not a consistent finding in either typical or atypical progeria syndromes. Abnormal calcification was previously proven to be part of the phenotype of HGPS. However, this condition includes a different disease mechanism, where the truncated and permanently farnesylated lamin A protein, progerin, is what accumulates in the cells, differently than LCPS. A study that performed RNAseq analysis of HGPS and healthy control cell lines showed that 911 transcripts were differentially expressed between healthy versus HGPS cell lines, including ITPR1, ITPR3, CACNA2D1, and CAMK2N1 linked to the calcium signaling pathway. These findings were orthogonally validated by Western blot analysis. It was observed that the basal concentrations of intracellular Ca^2+^ and reactive oxygen species were also statistically higher in HGPS cell lines compared to healthy ones [19]. Mice HGPS models *Lmna*G609G/+ were also proven to develop excessive aortic calcification similar to HGPS patients [17]. These studies noted a reduction in extracellular pyrophosphate levels in HGPS mice due to impaired synthesis of this product resulting from an up-regulation of the ectoenzymes TNAP (the main enzyme involved in pyrophosphate degradation) and eNTPD1 (an enzyme that hydrolyzes ATP to release phosphate) and reduced ATP production caused by reduced complex IV COX activity [20,21]. The discovery of this pathway might result in new treatment modalities for these patients, as it was previously demonstrated that a combination of the TNAP inhibitor levamisole and the eNTPD inhibitor ARL67156 prevented vascular calcification and extended longevity by 12% in HGPS mice [21]. Despite recent developments in the field, the understanding of the pathogenetic role of Lamin A/C in cardiac disease is still incomplete, as, for example, LCPS does not accumulate prelamin. Further studies are necessary to understand if the biochemical abnormalities demonstrated in HPS are also true for APS, including the different cellular processes affected by variants in this gene [3].

Currently, no curative treatment is available for any type of laminopathy; however, gene therapy approaches are currently under evaluation [21]. Pre-clinical studies exploring the therapeutic potential of different compounds that modulate the mammalian target of rapamycin kinase (mTOR) pathway, the mitogen-activated protein kinase (MAPK) cascade, and the epigenetic regulator N-acetyltransferase 10 (NAT10) have also been reported [3,22,23]. These studies illustrate how diverse therapeutic approaches might be for laminopathies, corroborating the need to better characterize the effects of different disease-associated variants for a tailored approach.

## 5. Conclusions

Testing for pathogenic variants in *LMNA* should be considered for patients presenting with marked calcification of many cardiac structures, including mitral and aortic valves. Our findings suggest that p.Glu2Lys affects nuclear morphology in a manner comparable to what was described in p.Asp300Gly cells. Characterizing the atypical forms of *LMNA*-linked progeria beyond prelamin accumulation is important for understanding the clinical course of these patients and for offering better treatment approaches when more therapies become available.

## Figures and Tables

**Table 1:** Oligonucleotide used in the generation of Lamin A-mRFP constructs.

Oligonucleotides
**Oligo 1 sequence:** ATGTGACGAGCTTCATTTATATCCTTCGCGCGCCGGACCGGCCTCCACAACTCGAGCTCAAGCTTCGAATTCTGCAGTCGAC GGTACCGCGGGCCCGGGATCCGAATGGCCATGGAGACCCCGTCCCAGCGGCGCGCCAATAGAGGCCAAGTTCGATTCGTAC TCCGATGTACGATACAACAATGTGG
**Oligo 2 sequence (p.E2K; c.4G>A):** ATGTGACGAGCTTCATTTATATCCTTCGCGCGCCGGACCGGCCTCCACAACTCGAGCTCAAGCTTCGAATTCTGCAGTCGA CGGTACCGCGGGCCCGGGATCCGAgccaccATGAAGACCCCGTCCCAGCGGCGCGCCAATAGAGGCCAAGTTCGATTCGTACTCCG ATGTACGATACAACAATGTGG
**Oligo 3 sequence (p.D300G; c.899A>G):** GCCCGCCTGCAGCTGGAGCTGAGCAAAGTGCGTGAGGAGTTTAAGGAGCTGAAAGCGCGCAATACCAAGAAGGAGGGTG ACCTGATAGCTGCTCAGGCTCGGCTGAAGGACCTGGAGGCTCTGCTGAACTCCAAGGAGGCCGCACTGAGCACTGCTCTC AGTGAGAAGCGCACGCTGGAGGGCGAGCTGCATGATCTGCGGGGCCAGGTGGCCAAGCTTGAGGCAGCCCTAGGTGAGG CCAAGAAGCAACTTCAGGATGAGATGCTGCGGCGGGTGGATGCTGAGAACAGGCTGCAGACCATGAAGGAGGAACTGGA CTTCCAGAAGAACATCTACAGTGAGGAGCTGCGTGAGACCAAGCGCCGTCATGAGACCCGACTGGTGGAGATTGACAATG GGAAGCAGCGTGAGTTTGAGAGCCGGCTGGCGGATGCGCTGCAGGAACTGCGGGCCCAGCATGAGGACCAGGTGGAGCA GTATAAGAAGGAGCTGGAGAAGACTTATTCTGCCAAGCTGGACAATGCCAGGCAGTCTGCTGAGAGGAACAGCAACCTGG TGGGGGCTGCCCACGAGGAGCTGCAGCAGTCGCGCATCCGCATCGGCAGCCTCTCTGCCCAGCTCAGCCAGCTCCAGAAG CAGCTGGCAGC

## Data Availability

The authors confirm that the data supporting the findings of this study are available within the article. The variants generated and/or analyzed during the current study are available in the LOVD (Leiden Open Variation Dataset) repository, Individual #00436422.

## Abbreviations

APS	Atypical progeria syndrome
HGP	Hutchinson–Gilford progeria
LADs	Lamina-associated domains
LCPS	*LMNA*-associated cardiocutaneous progeria syndrome
TAVR	Transcatheter aortic valve replacement
